# Investigating the effects of autonomy-supportive parenting practices on Italian young adolescent students’ motivation to defend victims of bullying: findings on the mediating roles of reactance, depression, anxiety, and stress

**DOI:** 10.3389/fpsyg.2023.1156807

**Published:** 2023-04-20

**Authors:** Nathaniel Oliver Iotti, Damiano Menin, Claudio Longobardi, Tomas Jungert

**Affiliations:** ^1^Department of Psychology, Lund University, Lund, Sweden; ^2^Department of Human Studies, University of Ferrara, Ferrara, Italy; ^3^Department of Psychology, Turin University, Turin, Italy

**Keywords:** parenting, bystander behavior, bullying, autonomy support, reactance

## Abstract

This study investigated the effect of autonomy-supportive parenting practices on young adolescents’ self-reported motivation to defend victims of bullying, and the possible mediating effects of factors such as reactance, anxiety, depression, and stress.

**Methods:**

Data were collected from 578 Italian public school students ages 10–14 (M_*age*_ = 11.8 years, 52% boys), who completed a survey in their classroom. The survey included self-report measures of parental orientation, motivation to defend victims of bullying, reactance, anxiety, depression, and stress.

**Results:**

We found that autonomy-supportive parenting had a positive effect on autonomous motivation to defend, and that this effect was weakly mediated by reactance. Moreover, autonomy-supportive parenting had a negative effect on extrinsic motivation to defend, which was partially mediated by reactance. Reactance had a positive direct effect on extrinsic motivation to defend, but results also showed that anxiety, depression, and stress did not mediate the effect of autonomy-supportive parenting on motivation to defend. Additionally, autonomy-supportive parenting appeared to play a protective role, being associated with lower levels of reactance, anxiety, depression, and stress. Finally, gender differences were found in our sample, with extrinsic motivation to defend being more prevalent in boys, and autonomous motivation to defend being more prevalent in girls. Girls also reported higher levels of anxiety, depression, and stress, compared to boys.

**Conclusion:**

Our findings show that autonomy-supportive parenting practices play a significant role in fostering young adolescents’ motivation to defend victims of bullying, and that they are also linked with lower feelings of reactance, anxiety, depression, and stress. We argue that interventions aimed at contrasting bullying and cyberbullying among youths should seek to involve parents more and promote the adoption of more autonomy-supportive parenting practices, due to their consistently proven beneficial effects.

## 1. Introduction

Bullying is defined as the intentional and repeated aggression of victims who cannot easily defend themselves (Olweus, 1993). A recent report by the Italian National Institute of Statistics (ISTAT, 2020) has highlighted that more than 50% of students ages 11–17 reported having been subjected to some form of bullying during the previous 12 months. Furthermore, almost 20% of students reported being bullied once or more per month, and, in 9% of these cases, the bullying had been a weekly occurrence. The literature has shown extensively that being bullied significantly affects the mental and physical health of victims, who may develop a wide range of internalizing and externalizing problems (Gini and Pozzoli, 2009; Reijntjes et al., 2010, 2011; Ortega et al., 2012; Hinduja and Patchin, 2019). Cyberbullying is bullying that is carried out through electronic means (e.g., social media, online games), and there is usually less emphasis on the conditions of repetition and power difference (Slonje et al., 2013; Brighi et al., 2019). This is because a single act of cyberbullying can have lasting consequences, as the material can be shared many times before it can be taken down. Additionally, power differences in cyberbullying might involve a difference in IT skills more than a difference in physical strength or popularity among peers. According to the previously mentioned survey ISTAT (2020), 22% of victims had been cyberbullied, and in 6% of cases the episodes had occurred several times per month. Cyberbullying is one of the most common forms of interpersonal violence among teenagers (Smith et al., 2008), and it has gathered much attention at various levels (e.g., Swearer et al., 2017; UNESCO, 2017; Abidin, 2019; Hinduja and Patchin, 2022).

Episodes of bullying and cyberbullying often involve bystanders, who can take on a variety of roles: from defending the victims, to remaining passive, to supporting or joining in the bullying (Salmivalli et al., 1996). Encouraging bystanders to defend victims is important because this has been found to reduce bullying episodes significantly (Kärnä et al., 2011; Salmivalli et al., 2011; Nocentini et al., 2013). However, defending victims of bullying and cyberbullying is not a completely risk-free endeavor: for example, individuals might fear retaliation, or a reduction of social status (Thornberg et al., 2012; Pepler et al., 2021). Therefore, bystanders rarely act as defenders (e.g., Craig et al., 2000), and it is essential to advance research into which factors could affect their motivation to defend victims, if we wish to support and encourage these behaviors, and use this knowledge to design better interventions. Jungert et al. (2016, 2021), have found that autonomous motivation, as conceptualized by Self-Determination Theory (SDT; Deci and Ryan, 2000), was positively associated with defending victims among Italian and Swedish adolescents. They also found that warm and supportive student-teacher relationships influenced adolescents’ motivation to defend victims (Jungert et al., 2016). However, to our knowledge there remains a need to investigate whether other meaningful adults in adolescents’ lives (e.g., parents/guardians, sports coaches, etc.) might affect their motivation to defend victims of bullying.

Notably, research has shown that parents are important in decreasing school bullying as they shape the development of bullying behavior in their children (Ahmed and Braithwaite, 2004). A couple of studies have found that children who experience their parents as authoritarian are involved in more bullying, while an authoritative style is linked to less bullying (Dehue et al., 2012), and that adolescents with authoritarian parents were at higher risk of cyberviolence (Moreno-Ruiz et al., 2018). Authoritarian parenting is characterized by low emotional warmth/support and high control/discipline, while authoritative parenting is characterized by high warmth/support and high control/discipline (Baumrind, 1968, 2012). The literature also describes permissive (high warmth/support, low control/discipline) and neglectful (low warmth/support, low control/discipline) parenting styles (Maccoby and Martin, 1983; Smetana, 2017). Attention was also given to the role of structure, level of involvement, and disciplinary practices in parenting, with structured environments, where parents give clear, logical, and predictable rules for their children to follow, proving to be more effective in encouraging and reinforcing positive behaviors, compared to more chaotic environments (Grolnick and Ryan, 1989; Smetana, 2017). Autonomy support in parenting is a concept that is closely intertwined with the previously mentioned dimensions of control/discipline, support, and structure that emerged in the literature, and it has been defined as “the degree to which parents value and use techniques which encourage independent problem solving, choice, and participation in decisions versus externally dictating outcomes, and motivating achievement through punitive disciplinary techniques, pressure, or controlling rewards” (Grolnick and Ryan, 1989, p. 144). Autonomy-supportive parenting has been found to be more effective in influencing the engagement of youths in adaptive or maladaptive behaviors, compared to controlling parenting, which entails the use of conditional affection and regard, as well as feelings of guilt, shame, and threats of punishment to modify children’s behaviors (Soenens et al., 2009; Soenens and Vansteenkiste, 2010; Ryan and Deci, 2017; Legate et al., 2019). Moreover, Legate et al. (2019) found that parents with an autonomy-supportive parenting style were less likely to have children who acted as cyberbullies than children of parents with a controlling style. Furthermore, autonomy-supportive parenting appears to support increased engagement in prosocial behaviors (Gagné, 2003), while controlling parenting has been linked with more aggressive and antisocial behaviors among children ages 6–12 (Joussemet et al., 2008). Finally, parenting styles are not a one-way factor that influences children and adolescents’ behavior, and the issue of feelings of reactance elicited by controlling parents provides a meaningful example of how certain practices might ultimately lead to an increase in the behavior they had originally tried to reduce, presumably because children view certain messages as invasive and oppressive (Legault et al., 2011; Bjelland et al., 2015). However, Legate et al. (2019) were not able to fully confirm these findings in their study, as they found that autonomy-supportive–but not controlling–parenting was related to adolescent reactance. As argued by the researchers themselves, these findings hint that there are measurement and conceptual issues underlying our knowledge and definitions of controlling and autonomy-supportive parenting styles, which must be addressed by future research, particularly concerning our understanding of controlling parenting. Overall, there is agreement among scholars that parenting practices have very significant effects on children’s wellbeing and socialization (Maccoby and Martin, 1983; Georgiou, 2008; Hinduja and Patchin, 2022). However, while parental autonomy support has been shown to influence children and youths’ motivation on issues such as academic performance (Banerjee and Halder, 2021), pro-environmental behavior (Grønhøj and Thøgersen, 2017), and dieting (Katz et al., 2015), we still do not know whether it might also play a role in influencing children’s motivation to defend victims of bullying.

In the present study, we investigated whether young adolescents with parents who adopted more autonomy-supportive strategies when regulating their unwanted behaviors would exhibit a higher autonomous motivation to defend victims. Additionally, we investigated the possible mediating roles of reactance and factors such as depression, anxiety, and stress between autonomy-supportive parenting and motivation to defend, as well as controlling for possible gender differences. This was decided because the available evidence on gender differences is inconclusive, with some studies on prosocial motivation reporting that girls exhibit more autonomous motivation than boys (e.g., Vansteenkiste et al., 2009), while other studies were unable to confirm the presence of said differences (e.g., Iotti et al., 2022). Furthermore, we wanted to investigate possible gender differences in internalizing symptoms, considering the compelling evidence on the presence of a gender gap in mental health between girls and boys (Torsheim et al., 2006; Campbell et al., 2021). This study stems from a larger project, which was pre-registered on the Open Science Framework on November^1^ 14th, 2021.

## 2. Materials and methods

### 2.1. Participants and procedure

Our sampling procedure was based on convenience and availability. It should be mentioned that several schools that were contacted initially did not agree to participate in the study because of time and organizational constraints, mostly brought on by a particularly difficult return to normal routines after the SARS-CoV-2 global pandemic. We were therefore able to select 32 classes from five middle schools in the Metropolitan area of Turin, Italy. The study was approved by the university’s ethical review board (prot. no. 291035). Principals, teachers, parents/guardians, and the students themselves expressed written consent to participate, after being informed about the study and of their right to refuse or withdraw their consent at any time, in compliance with the ethical code of the Italian Association for Psychology (AIP) and the Declaration of Helsinki. The final sample consisted of 578 students ages 10–14 years (M = 11.8, sd = 0.79 years, 52% male). The students completed a survey, which included an adapted version of the General Causality Orientation Scale (Deci and Ryan, 1985; Legate et al., 2019), a reactance scale (Vansteenkiste et al., 2014), the Depression, Anxiety, and Stress Scale (Lovibond and Lovibond, 1995), and the Motivation to Defend Scale (Jungert et al., 2016; Iotti et al., 2022), in their classrooms during school hours. Research assistants were present during data collection to aid participants and provide clarifications when needed. The survey took approximately 20–30 min to complete, and participants did not receive any form of retribution or compensation for their involvement in the study.

### 2.2. Measures

#### 2.2.1. Autonomy-supportive parenting

Autonomy-supportive parenting was measured with the General Causality Orientation Scale (GCOS; Deci and Ryan, 1985; Legate et al., 2019), which is an instrument that assesses parental tendencies to use autonomy-supportive vs. controlling parenting styles. It consists of 10 hypothetical bullying/cyberbullying scenarios (e.g., “Your parent just found out you have been using social media to post insulting messages about a schoolmate” or “Your parent noticed you went out of your way to include a shy classmate in a peer group”) in which adolescents are presented with two possible reactions to the scenario, one autonomy-supportive and one controlling, and they are asked to express how likely it is that their parents would react in that manner. Each item is answered on a 6-point Likert scale (1 = *Very unlikely*, 6 = *Very likely*). The original scale was developed in English, and was translated into Italian for the purposes of this study following Van de Vijver and Hambleton’s (1996) established guidelines.

#### 2.2.2. Motivation to defend

Motivation to defend was measured with the Motivation to Defend Scale (MDS; Jungert et al., 2016; Iotti et al., 2022), which is a self-report instrument designed for young adolescents (ages 10–15 years). It measures a child’s motivation to defend victims of bullying on four subscales: Intrinsic (e.g., “Because I like to help other people”), Identified (e.g., “Because I think it is important to help people who are treated badly”), Introjected (e.g., “Because I would feel like a bad person if I did not try to help”), and Extrinsic (e.g., “In order to receive a reward”). Participants are asked to think of situations where they had witnessed other students being bullied and to report why they would engage in helping someone who is a victim of bullying. Each of the 16 items is answered on a 5-point Likert scale (1 = *Completely disagree*, 5 = *Completely agree*). In line with previous research (Jungert and Perrin, 2019; Iotti et al., 2022), we used the Intrinsic and Identified subscales to calculate Autonomous motivation.

#### 2.2.3. Depression, anxiety and stress

These factors were measured with the Depression, Anxiety and Stress Scale (DASS-21; Lovibond and Lovibond, 1995; Bottesi et al., 2015), which is a self-report 21-item instrument that is widely used to measure stress, anxiety and depression in children and adolescents. It is divided into three scales, which contain seven items each, and are divided into subscales with similar content. The Depression scale assesses dysphoria, hopelessness, devaluation of life, self-deprecation, lack of interest/involvement, anhedonia, and inertia. The Anxiety scale assesses autonomic arousal, skeletal muscle effects, situational anxiety, and subjective experience of anxious affect. The Stress scale assesses difficulty relaxing, nervous arousal, and being easily upset/agitated, irritable/over reactive, and impatient. Participants are asked to indicate on a 4-point Likert scale (1 = *Did not apply to me*, 4 = *Applied to me very much, or most of the time*) how much each of the 21 statements applied to them over the past week.

#### 2.2.4. Reactance

Finally, we adapted seven items taken from the literature (Vansteenkiste et al., 2014; Legate et al., 2019) to measure adolescents’ feelings of reactance toward the target parent regulating their social behavior. First, participants read the following prompt: “When my (target parent) wants me to act in a certain way (e.g., being nice to others on social media), these conversations…” and then they are asked to express their level of agreement with a series of statements (e.g., “Make me think that I want to do exactly the opposite”). Each item is answered on a 5-point Likert scale (1 = *Strongly disagree*, 5 = *Strongly agree*). The original scale was developed in English, and was translated into Italian for the purposes of this study following Van de Vijver and Hambleton’s (1996) established guidelines.

### 2.3. Analysis plan

We used Confirmatory Factor Analysis (CFA) to assess the goodness of the factor structure, and Structural Equation Modeling (SEM) to test a series of models that included parenting style as the exogenous variable, reactance, depression, anxiety, and stress as mediators, and autonomous and extrinsic motivation to defend as outcomes, using the distributionally-robust weighted least square mean and variance adjusted estimator (WLSMV). All analyses were carried out in the R statistical environment, version 4.2.2, using the lavaan package (Rosseel, 2012).

## 3. Results

We performed a CFA to test the main measurement model, and it showed a good fit: CFI = 0.963, TLI = 0.958, RMSEA = 0.050, SRMR = 0.053. Items with loadings lower than 0.4 were removed (i.e., item 10 from the autonomy-supportive parenting GCOS scale, item 5 on the Extrinsic MDS subscale, and items 5–7 on the reactance scale). Factor loadings were all significantly different from zero with *p* < 0.001 (see Table 1).

**TABLE 1:** Confirmatory Factor Analysis (CFA) covariance matrix.

	EXM	AUM	REA	ASP
Extrinsic motivation (EXM)	–	−0.511***	0.350***	-0.357***
Autonomous motivation (AUM)	–	–	-0.262***	0.523***
Reactance (REA)	–	–	–	-0.347***
Autonomy-supportive parenting (ASP)	–	–	–	–

***p < 0.001.

### 3.1. Reactance-only model

**FIGURE 1:**
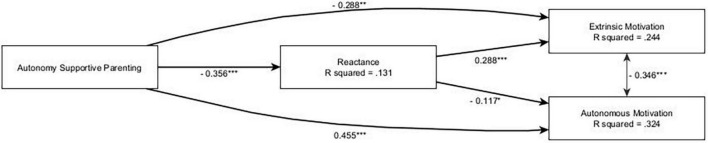
The reactance-only main model. ***p < 0.001; **0.001 < *p* < 0.01; *0.01 < *p* < 0.05; only significant effects (*p* < 0.05) are shown.

We fitted our main model with reactance as the sole mediator, parenting style and gender as exogenous variables, and motivation to defend as outcomes, and it showed a good fit: CFI = 0.962, TLI = 0.960, RMSEA = 0.048, SRMR = 0.054. SEM analysis (see Figure 1) highlighted a positive total effect of autonomy-supportive parenting on autonomous motivation (β = 0.496, *p* < 0.001), which was only weakly mediated by reactance (β = 0.042, *p* = 0.040). In particular, autonomy-supportive parenting had a negative effect on reactance (β = −0.356, *p* < 0.001), which in turn had a negative effect on autonomous motivation (β = −0.117, *p* = 0.041). Conversely, autonomy-supportive parenting had a negative total effect on extrinsic motivation (β = −0.331, *p* < 0.001), which was partially mediated by reactance (β = −0.103, *p* < 0.001). Additionally, reactance was positively associated with extrinsic motivation (β = 0.288, *p* < 0.001). Furthermore, the model highlighted some gender differences, with autonomous motivation being more prevalent in females (β = 0.265, *p* < 0.001), and extrinsic motivation being more prevalent in males (β = −0.267, *p* < 0.001).

### 3.2. Anxiety, depression, and stress alternative models

Subsequently, to answer our question of whether psychological factors such as depression, anxiety or stress might also contribute to mediate the effect of autonomy-supportive parenting on motivation to defend, and to explore their possible relationship with reactance, we fitted three separate models with reactance and either anxiety, depression, or stress as mediators, while maintaining the exogenous variables and outcomes of the previous main model. The results are reported below:

#### 3.2.1. Anxiety

**FIGURE 2:**
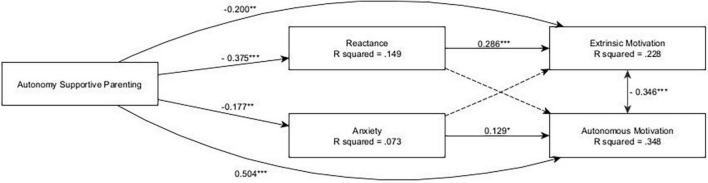
The anxiety and reactance alternative model. ***p < 0.001; **0.001 < *p* < 0.01; *0.01 < *p* < 0.05; only significant effects (*p* < 0.05) are shown.

The model showed an acceptable fit: CFI = 0.940, TLI = 0.938, RMSEA = 0.038, SRMR = 0.053. SEM analysis (see Figure 2) highlighted a positive total effect of autonomy-supportive parenting on autonomous motivation (β = 0.510, *p* < 0.001), which was not mediated by anxiety or reactance. Specifically, autonomy-supportive parenting was negatively associated with anxiety (β = −0.177, *p* < 0.001) and reactance (β = −0.375, *p* < 0.001), while anxiety showed only a small effect on autonomous motivation (β = 0.129, *p* = 0.020), and reactance did not have a significant effect on autonomous motivation. Conversely, autonomy-supportive parenting had a negative total effect on extrinsic motivation (β = −0.307, *p* < 0.001), which was partially mediated by reactance (β = −0.107, *p* = 0.001), which had a positive effect on extrinsic motivation (β = 0.286, *p* < 0.001), but not by anxiety. Furthermore, the model highlighted some gender differences, with autonomous motivation being more prevalent in females (β = 0.239, *p* < 0.001), extrinsic motivation being more prevalent in males (β = −0.274, *p* < 0.001), and females reporting higher levels of anxiety (β = 0.204, *p* < 0.001).

#### 3.2.2. Depression

**FIGURE 3:**
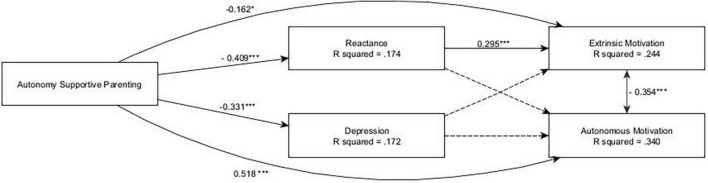
The depression and reactance alternative model. ***p < 0.001; *0.01 < *p* < 0.05; only significant effects (*p* < 0.05) are shown.

The model showed an acceptable fit: CFI = 0.891, TLI = 0.887, RMSEA = 0.050, SRMR = 0.063. SEM analysis (see Figure 3) highlighted a positive direct effect of autonomy-supportive parenting on autonomous motivation (β = 0.518, *p* < 0.001), which was not mediated by depression or reactance. Specifically, autonomy-supportive parenting was negatively associated with depression (β = −0.331, *p* < 0.001) and reactance (β = −0.409, *p* < 0.001), while neither reactance nor depression showed any effects on autonomous motivation. Conversely, autonomy-supportive parenting had a negative total effect on extrinsic motivation (β = −0.283, *p* < 0.001), which was partially mediated by reactance (β = −0.121, *p* = 0.001), which had a positive effect on extrinsic motivation (β = 0.295, *p* < 0.001), but not by depression. Furthermore, the model highlighted some gender differences, with autonomous motivation being more prevalent in females (β = 0.253, *p* < 0.001), extrinsic motivation being more prevalent in males (β = −0.306, *p* < 0.001), and females reporting higher levels of depression (β = 0.249, *p* < 0.001).

#### 3.2.3. Stress

**FIGURE 4:**
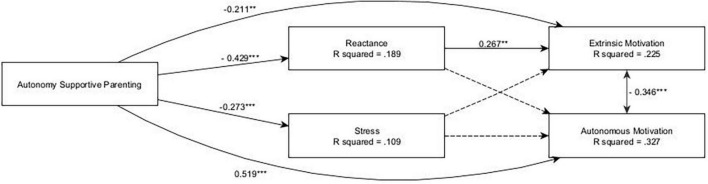
The stress and reactance alternative model. ***p < 0.001; **0.001 < *p* < 0.01; only significant effects (*p* < 0.05) are shown.

The model showed an acceptable fit: CFI = 0.896, TLI = 0.892, RMSEA = 0.049, SRMR = 0.064. SEM analysis (see Figure 4) highlighted a positive direct effect of autonomy-supportive parenting on autonomous motivation (β = 0.519, *p* < 0.001), which was not mediated by stress or reactance. Specifically, autonomy-supportive parenting was negatively associated with stress (β = −0.273, *p* < 0.001) and reactance (β = −0.429, *p* < 0.001), while neither reactance nor stress showed any effects on autonomous motivation. Conversely, autonomy-supportive parenting had a negative total effect on extrinsic motivation (β = −0.326, *p* < 0.001), which was partially mediated by reactance (β = −0.115, *p* = 0.002), which had a positive effect on extrinsic motivation (β = 0.267, *p* = 0.001), but not by stress. Furthermore, the model highlighted some gender differences, with autonomous motivation being more prevalent in females (β = 0.247, *p* < 0.001), extrinsic motivation being more prevalent in males (β = −0.265, *p* < 0.001), and females reporting higher levels of stress (β = 0.184, *p* < 0.001).

## 4. Discussion

### 4.1. Findings from the main model

Autonomy-supportive parenting was found to have a positive effect on autonomous motivation to defend, and a negative effect on extrinsic motivation to defend. Both effects were mediated by reactance, and the proportion mediated (PM) was higher for the effect on extrinsic motivation (β = 0.312), while just 8.5% of the total effect of autonomy-supportive parenting on autonomous motivation was found to be mediated by reactance (β = 0.085). Therefore, we were able to confirm previous findings that reactance partially contributes to explain the effect of autonomy-supportive parenting practices on youths’ behavior and motivation (Legate et al., 2019), with particular regard to autonomous motivation. According to SDT, a distinction can be made between intrinsic motivation, which entails carrying out an action because of interest or enjoyment, and extrinsic motivation, which entails carrying out an action exclusively to obtain a separable outcome, such as fame or money (Ryan and Deci, 2000). Intrinsic motivation is considered to be more stable and preferable, when possible, than extrinsic motivation because it is not dependent on external factors and contingencies (Deci and Ryan, 2000). Autonomous motivation to defend, as conceptualized in this study, is a form of intrinsic motivation because it involves defending victims voluntarily, and not out of a desire for rewards, fame, or fear of punishment. Furthermore, autonomous motivation to defend is characterized by a desire to help both because the agents report personal enjoyment in carrying out these kinds of actions (e.g., “Because I like to do those kinds of things”), or because they attribute value to the notion of helping (e.g., “Because I think it’s important to help people who are treated badly”), but also because they support and identify with the idea of fighting violence and injustice in general. As mentioned previously, autonomous motivation to defend has been associated with increased defending behaviors (Jungert et al., 2016, 2021; Longobardi et al., 2020), as well as with stronger persistence and performance in a wide range of activities and behaviors (e.g., Niemiec and Ryan, 2009; Moran et al., 2012; Taylor et al., 2014). Therefore, our finding that autonomy-supportive parenting appears to increase autonomous motivation to defend, and lower extrinsic motivation to defend confirms the existing evidence on the positive effect of autonomy-supportive practices on bystanders’ motivation to defend victims of bullying (Jungert et al., 2016). Moreover, it supports the existing evidence on the positive role played by parental autonomy support in influencing youths’ motivation in a variety of behaviors (Katz et al., 2015; Grønhøj and Thøgersen, 2017; Banerjee and Halder, 2021). Additionally, our finding that autonomy-supportive parenting elicits less reactance in young adolescents is in line with the evidence available in the literature (Legate et al., 2019), and supports the theory that practices based on providing clear and logical behavioral expectations, and that respect youths’ perspectives and agency, elicit less feelings of reactance because they are perceived as less invasive, and youths are less likely to engage in oppositional behaviors to contrast them (Assor et al., 2004; Ryan and Deci, 2017). Finally, our findings concerning the effects of reactance on extrinsic and autonomous motivation to defend are in line with the existing SDT research (Ryan and Deci, 2017) because extrinsic motivation, which is by definition more reliant on contingencies, was expected to be influenced by factors such as reactance to a greater degree, compared to autonomous motivation, which does not depend on external factors, and was only weakly negatively affected by reactance in our sample.

### 4.2. Findings from the alternative model and gender differences

Our results also showed that, while autonomy-supportive parenting had a protective effect on mental wellbeing, being associated with lower levels of depression, stress, and anxiety, none of these factors contributed to explaining the effect of autonomy-supportive parenting on motivation to defend. Furthermore, depression and stress were not found to have a direct effect on either autonomous or extrinsic motivation to defend, and anxiety was found to have only a small positive effect on autonomous motivation to defend. These findings might appear to be somewhat counterintuitive at a first glance, but they could be explained in the following manner: because autonomous motivation does not depend on contingencies, it is less affected by factors such as depression or stress, and previous studies have found both negative and positive associations between anxiety and autonomous motivation to defend (Jungert and Perrin, 2019; Jungert et al., 2021), which are believed to depend on the type of bullying being witnessed, and to support the theory that bystanders’ emotional states influence their willingness to intervene (Fischer et al., 2011; Hortensius and De Gelder, 2018). However, according to our reasoning, extrinsic motivation to defend should have been more susceptible to factors such as anxiety, depression, and stress, and this was not the case. A possible explanation for this finding is that our sample did not present high or pathologic rates of any of these factors, and this could have affected our results. It is possible that different results might be found in samples with higher rates of depression, stress, and anxiety. However, an alternative explanation is that, because of the way extrinsic motivation to defend is currently conceptualized, it is more influenced by behavioral factors such as reactance than by psychological factors such as anxiety, depression, or stress, which are more likely to influence other forms of external regulation, such as introjected motivation, which were not considered in this study (Ryan and Deci, 2000). Nonetheless, our finding that autonomy-supportive parenting appeared to lower depression, stress, and anxiety, is in line with the existing evidence on the protective effect of caring and supportive parenting strategies on youths’ mental wellbeing (e.g., Ortega et al., 2021; Rakhshani et al., 2022). Finally, the gender differences that emerged in our sample could be explained by some evidence in motivational research of girls showing more autonomous motivation than boys (Ratelle et al., 2007; Vansteenkiste et al., 2009), but findings of gender differences in prosocial motivation have not been confirmed by other studies (Jungert et al., 2021; Iotti et al., 2022). Moreover, girls in our sample reported higher levels of anxiety, depression, and stress, compared to boys, in line with the current literature on gender differences in mental health, which reports that girls have worse internalizing mental health than boys, and that this gender gap continues to increase with age (Cavallo et al., 2006; Torsheim et al., 2006; Campbell et al., 2021).

### 4.3. Limitations and future directions

Some limitations of the study must be addressed. First, we used self-report measures, which are sensitive to shared methods variance effects, and to a series of biases, such as those of recall, social desirability, and perception. However, we argue that, when assessing parenting practices, children might be less vulnerable to social desirability or other biases, compared to their parents. Additionally, because we were not able to secure parent-child dyads for this study and we had to rely on child-reports only, we could not exclude the likelihood of effects resulting from third factors or alternative causal pathways completely. When possible, future studies should consider integrating behavioral or observational methods in their coding of parenting styles, and enrolling parent-child dyads as a partial solution to contrast these issues. Furthermore, studies that use qualitative or mixed-methods approaches would be advisable in the future, as there is a need to integrate our current knowledge with youths’ own accounts and perceptions of parenting practices and how they relate with their behavior and motivation. Second, the adoption of a cross-sectional study design does not allow us to fully determine the direction of effects between the variables or exclude the possibility of bidirectional effects. Therefore, future studies should use longitudinal designs to contrast these issues and provide information on the effect of parenting styles on youth’s motivation through time. Finally, due to how our sample was selected, our results might not be fully representative of the Italian young adolescent population and would benefit from future replication in other regions, countries, and populations.

## 5. Conclusion

Our study provides promising evidence on the effect of autonomy-supportive parenting practices on youths’ motivation to defend victims of bullying, and on the internal factors that influence this relationship, improving current knowledge of the individual and contextual factors that influence bystander behaviors in bullying episodes. Our findings also suggest that interventions aimed at reducing bullying, and perhaps other problem behaviors among young adolescent populations, should involve parents to a greater extent and help them adopt more autonomy-supportive practices because of their effect on youth’s motivation to defend victims, their capacity to elicit lower feelings of reactance toward behavior regulation attempts, and their overall positive effect on mental health. Specifically, girls might experience increased benefits from their parents adopting more autonomy-supportive practices exactly because of their positive association with mental health. In conclusion, because parents have a profound and lasting influence on their children’s motivation, behaviors, and mental health, it is important to involve them in bullying and cyberbullying research and prevention, and to help them adopt practices that are more effective and supportive of youths’ autonomy and wellbeing.

## Data availability statement

The raw data supporting the conclusions of this article will be made available by the authors, without undue reservation.

## Ethics statement

The studies involving human participants were reviewed and approved by University of Turin Bioethics Committee (Comitato Bioetico dell’Ateneo dell’Universita’ degli Studi di Torino). Written informed consent to participate in this study was provided by the participants and the participants’ legal guardian/next of kin.

## Author contributions

NI: methodology, formal analysis, and writing the first draft of the manuscript. DM: methodology, carrying out the data curation and formal analysis, and writing sections of the manuscript. TJ: methodology, overseeing the project supervision, and assisting with writing and editing the manuscript. CL: project supervision, overseeing the data collection, and assisting with editing the manuscript. All authors contributed to the conception and design of the study, final manuscript revisions, read, and approved the submitted version.

## Footnotes

1
https://osf.io/aeq3v
